# Transformer-Enhanced Localization via Adaptive PDP Representation Under Dynamic Bandwidths

**DOI:** 10.3390/s26051486

**Published:** 2026-02-27

**Authors:** Lei Cao, Tianqi Xiang, Weiyan Chen, Yicheng Wang, Yuehong Gao, Xin Zhang

**Affiliations:** 1 China Mobile Research Institute, Beijing 100080, China; 2 School of Information and Communication Engineering, Beijing University of Posts and Telecommunications, Beijing 100876, China; xiangtianqi@bupt.edu.cn (T.X.);

**Keywords:** learning-based localization, localization for IoT devices and AAV, neural network and Transformer

## Abstract

Accurate wireless positioning has remained challenging under dynamic bandwidth conditions and outdoor multipath environments that are typical in Internet of Things (IoT) and autonomous aerial vehicle (AAV) applications. Conventional learning-based localization methods rely on bandwidth-specific channel state information (CSI) representations, which causes the trained models to be inapplicable or less adaptive when the signal bandwidth differs from that used during training. To overcome this limitation, a unified and neural network-oriented framework is proposed, which constructs bandwidth-adaptive power delay profile (PDP) representations for learning-based models. A PDP preprocessing scheme through adaptive zero-padding and oversampled IFFT of heterogeneous CSI is introduced to generate dimension-consistent and delay-aligned neural network inputs. To enhance robustness, a sub-band-sliced PDP representation is developed to enhance model robustness, where each bandwidth is divided into equal-width sub-bands whose PDPs are independently processed and organized as Transformer tokens. A dedicated Transformer is designed to get the location estimation from PDPs of multi-access points. Simulation results have demonstrated that the proposed preprocessing-PDP-plus-Transformer framework achieves superior cross-bandwidth generalization and localization accuracy, compared to analytical and learning-based baselines.

## 1. Introduction

The rapid development of Aerial Autonomous Vehicles (AAVs) has accelerated the need for high-accuracy positioning, which is fundamental for autonomous flight control, collision avoidance, and environment-aware decision-making in complex and dynamic scenarios.

Conventional global navigation satellite systems (GNSS) could provide a widely available solution for outdoor positioning. However, GNSS suffered from several limitations, including degraded performance under multipath conditions near buildings or landing grounds, as well as vulnerability to occlusions [1]. Ultra-wide-band (UWB) tag-based positioning could achieve higher accuracy by exploiting large bandwidths and line-of-sight propagation [2]. A heterogeneous sensing system [3] was proposed to achieve AAV localization when GNSS is not available, which integrated a light scanner, UWB and an inertial navigation system. But these alternatives require dedicated anchors and additional payload, which may be impractical for lightweight or energy-constrained AAV systems.

As another alternative, recent researches [4] highlighted that wireless communication infrastructures, such as Wi-Fi access points and cellular base stations, could be leveraged as distributed radio sensors, which enabled ubiquitous and cost-effective localization capabilities. Classical approaches exploit physical-domain measurements derived from wireless signals, including time-of-arrival (TOA)/time-difference-of-arrival (TDOA) [5], and path-based geometric reconstruction [6]. To enhance delay resolution, super-resolution techniques such as MUSIC [7,8] and ESPRIT [9] have been explored. However, these analytical estimators remain fundamentally constrained by bandwidth-limited resolution [10].

More recently, data-driven localization approaches have emerged as powerful alternatives to traditional geometric and statistical methods [11]. Existing learning-based localization methods can be broadly categorized into three main classes.

Conventional CSI fingerprinting-based deep learning approaches directly exploit raw or lightly processed channel state information (CSI) as network inputs. Representative works employ fully connected networks [12], convolutional architectures [13,14,15,16], long short-term memory architectures [17], or phase-centric representations [18] to infer 2D or 3D user locations. While these methods can achieve high positioning accuracy under fixed system configurations, their input dimensionality is inherently tied to the system bandwidth and subcarrier configuration, making the trained models difficult to reuse or generalize when bandwidth resources change.

Another line of research focuses on learning-based localization using physically meaningful delay-domain features, such as time-of-arrival (TOA), time-difference-of-arrival (TDOA), received signal strength indicators (RSSI), or parameterized channel impulse responses (CIR) [19,20,21]. Approaches based on handcrafted physical features intentionally compress raw CSI into a small number of interpretable parameters, which simplifies learning and enables geometric modeling. However, this compression inevitably discards rich multipath information inherently contained in CSI, including fine-grained delay dispersion, interpath interference, and frequency-dependent amplitude–phase patterns. This information loss becomes particularly critical under limited bandwidth, where insufficient delay resolution prevents reliable separation of the LOS path from strong secondary reflections, causing severe bias in TOA estimation. In contrast, CSI or PDP-level representations preserve substantially more multipath structure and are therefore more suitable for data-driven localization frameworks that aim to exploit, rather than suppress, multipath effects.

With the recent success of attention mechanisms, Transformer-based architectures have been introduced into wireless localization [22,23,24]. By modeling long-range dependencies across antennas, subcarriers, or access points (APs), Transformers have demonstrated superior representation capability compared to conventional neural networks. Nevertheless, existing Transformer-based localization frameworks still rely on fixed-dimensional CSI or CIR inputs and do not explicitly address the challenge of dynamically varying bandwidth resources.

In summary, despite their respective advantages, existing data-driven localization methods generally face a trade-off between positioning accuracy and system flexibility. Feature-based approaches sacrifice information during preprocessing, while CSI- and CIR-based neural networks remain tightly coupled to specific bandwidth configurations, limiting parameter sharing and generalization under dynamic sensing resources [25]. This motivates the development of bandwidth-adaptive representations and learning frameworks that can simultaneously preserve rich channel information and support robust localization across heterogeneous bandwidth conditions. This work proposes power delay profile (PDP)-based preprocessing schemes and dedicated Transformer architectures, which enable reliable access point (AP)-based AAV localization across dynamic bandwidth resources. The main contributions of this paper are summarized as follows.

- We propose a bandwidth-independent preprocessing method based on zero-padded PDP (ZP-PDP). By adaptively inserting zeros to the CSI followed by a controlled truncation of the PDP, the resulting representation achieves a unified delay span and resolution regardless of the system bandwidth. A dedicated Transformer encoder architecture is further developed to process the multi-AP ZP-PDP measurements, which enables parameter sharing and high-accuracy localization under dynamically varying bandwidth resources.
- To enhance robustness across localization bandwidths, we introduce a sub-band-sliced PDP (SS-PDP) representation. By dividing the CSI into equal-width sub-bands, each sub-band naturally yields a PDP with aligned delay span and resolution. The resulting multi-sub-band PDPs are organized as a variable-length token sequence compatible with the Transformer’s flexible attention mechanism, which enables strong generalization to unseen bandwidths.
- We establish a comprehensive benchmarking framework that evaluates neural networks under dynamically changing bandwidth resources. The framework includes model-size analysis under different bandwidths, same-bandwidth testing, and cross-bandwidth inference performance. Numerical results show that both proposed PDP-based methods achieve accuracy comparable to or better than the CSI integrated with Transformer baseline, while the SS-PDP method delivers the strongest robustness in cross-bandwidth localization.

The rest of this paper is organized as follows. Section 2 describes the system model and problem formulation and indicates the constraints of existing CSI-based neural networks. Section 3 introduces the proposed ZP-PDP and SS-PDP methods and the corresponding Transformer encoder-based neural networks. Section 4 gives the numerical results and Section 5 concludes this paper.

## 2. System Model and Problem Formulation

### 2.1. System Model

**Figure 1:**
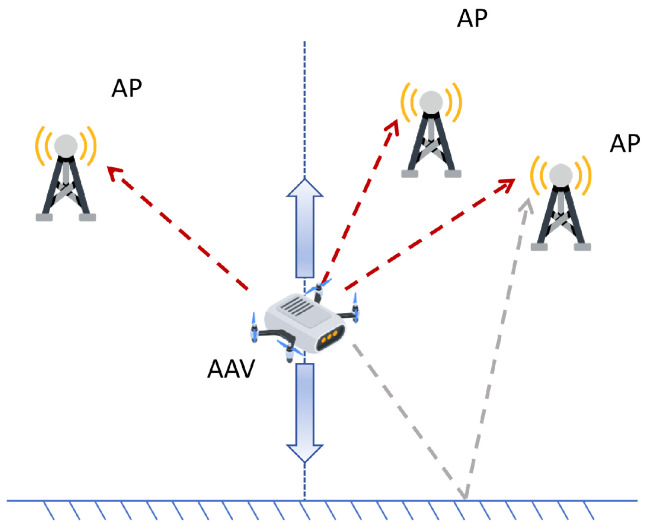
Illustration of AAV localization.

We consider a wireless sensing and communication scenario in which an AAV performs take-off and landing within a structured landing zone containing buildings, vegetation, metallic infrastructure, and other objects that give rise to rich and spatially varying multipath propagation. The scenario is illustrated in Figure 1. A set of $N_{\mathrm{AP}}$ APs is deployed around the area to support communication and localization services. Each AP is equipped with a single antenna. The AAV transmits uplink OFDM pilot signals, which are received at the AP for positioning purposes. The uplink design is consistent with practical deployments in which the computationally intensive localization algorithms, potentially including deep neural networks, are executed on the AP or an edge server with sufficient computing capability, while the AAV remains lightweight. The OFDM pilot occupies one of several possible system bandwidths. The AP acquires a single-shot frequency-domain channel measurement on *K* subcarriers. These measurements form the basis for the delay-domain or neural network-based localization methods which will be developed later.

Let an Aerial Autonomous Vehicle (AAV) transmit an uplink pilot symbol $s_{k}$ on subcarrier *k*. For the *n*-th AP, the received signal on subcarrier *k* is

(1) $$y_{n,k}\;=\;h_{n,k}\;s_{k}+n_{n,k},$$

where $n_{n,k}∼\mathrm{CN}\left(0,\;\sigma^{2}\right)$ denotes additive white Gaussian noise and $h_{n,k}$ is the uplink channel frequency response between the AAV and AP *n* on subcarrier *k*. The uplink channel on subcarrier *k* at AP *n* is modeled as a superposition of $L_{n}$ multipath components [26]:

(2) $$h_{n,k}=\sum_{\ell =1}^{L_{n}}\alpha_{n,\ell }\exp\;\left(-j2\pi f_{k}\tau_{n,\ell }\right),$$

where $\alpha_{n,\ell }$ is the complex gain of path *ℓ*, $\tau_{n,\ell }$ is its propagation delay, $f_{k}$ is the absolute frequency of subcarrier *k*, and $L_{n}$ denotes the number of resolvable paths for AP *n*.

### 2.2. Problem Formulation

Consider an AAV located at an unknown 3D coordinate $u={\left[x,\;y,\;z\right]}^{T}\in R^{3}$, and let there be $N_{\mathrm{AP}}$ access points (APs) deployed at known positions $b_{1},\;b_{2},\;\ldots ,\;b_{N_{\mathrm{AP}}}\in R^{3}$. During each uplink sounding instance, the AAV transmits a reference signal over a system bandwidth *B* with *K* subcarriers. Each AP *n* acquires a frequency-domain measurement vector

(3) $$y_{n}={\left[\;y_{n,1},\;y_{n,2},\;\ldots ,\;y_{n,K}\;\right]}^{T}\in C^{K},\;n=1,\ldots ,N_{\mathrm{AP}}.$$

By stacking the measurements from all APs, we yield the full observation

(4) $$Y={\left[y_{1},\;y_{2},\;\ldots ,\;y_{N_{\mathrm{AP}}}\right]}^{T}\in C^{K\times N_{\mathrm{AP}}}.$$

We consider uplink-based channel measurements acquired via a single sounding instance, such as an uplink Sounding Reference Signal (SRS), where multiple APs simultaneously estimate the frequency-domain channel response from the AAV. The resulting delay-domain representation corresponds to relative multipath delays rather than absolute TOA measurements. Therefore, absolute time synchronization between the AAV and APs is not required. We assume that APs are mutually synchronized in time and frequency, as commonly supported in 3GPP deployments through GNSS or IEEE 1588-based [27] mechanisms. Given the short duration of uplink sounding (on the order of milliseconds), the AAV displacement during one measurement snapshot is negligible, even during takeoff or landing.

Because the AAV operates close to the ground during takeoff and landing, the wireless channel under sub-6GHz or millimeter wave is dominated by a direct path and a ground-reflected path [28], whose delays and amplitudes are functions of the unknown AAV position u and the AP locations $b_{n}$. The objective is to learn a mapping,

(5) $$f_{\theta }:C^{K\times N_{\mathrm{AP}}}\to R^{3},$$

parameterized by neural network weights θ, such that

(6) $$\hat{u}=f_{\theta }\left(Y\right)$$

approximates the true AAV location u. Then supervised learning is performed by minimizing the localization error:

(7) $$\theta^{⋆}=\arg\min_{\theta }{∥f_{\theta }\;\left(Y\right)-u∥}_{2}^{2}.$$

A key challenge arises because the measurement vector dimension *K* varies with the available bandwidth *B*, causing the joint AP observation Y to have bandwidth-dependent dimensionality. This prevents direct parameter sharing across heterogeneous bandwidths and severely limits the generalization of neural networks trained under a single bandwidth configuration. This paper proposes PDP-based preprocessing schemes and corresponding Transformer-based architectures to construct bandwidth-independent representations, enabling accurate multi-AP localization even under dynamic or mismatched bandwidth conditions.

## 3. Transformer-Enhanced Localization via Bandwidth-Independent PDP

To address the challenge posed by bandwidth-dependent CSI dimensionality, we propose two complementary preprocessing–learning pipelines that construct bandwidth-independent representations of the uplink CSI. Both methods convert the raw multi-AP CSI into delay-domain power profiles (PDPs), which naturally encode propagation delays and multipath structure. The first method creates bandwidth-adaptive PDPs via zero padding with unified time delay scale; the second method constructs sub-band-sliced PDPs based on subdividing the available bandwidth. The resulting PDPs are then processed by dedicated Transformer architectures that exploit multi-AP PDP measurements.

It is worth noting that the delay-domain PDP representation does not require absolute time synchronization between the AAV and the APs. When multiple APs are mutually synchronized, the PDPs extracted at different APs preserve the relative multipath delay structure induced by the AAV location and the surrounding environment. Such relative delay information is sufficient for localization, either from a time-difference-based perspective or from a fingerprinting perspective that exploits multipath-induced spatial signatures. In this sense, the PDP serves as a physically meaningful representation that embeds both geometric distance information and environment-dependent multipath characteristics.

### 3.1. Bandwidth-Adaptive Zero-Padded PDP Representation Approach

**Figure 2:**
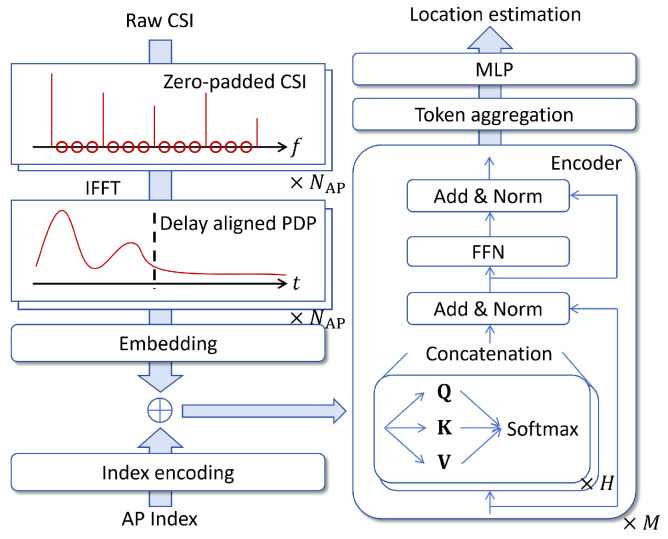
Illustration of the ZP-PDP approach.

In this scheme, to ensure that the feature representation remains consistent across bandwidths, we design a preprocessing zero-padded PDP (ZP-PDP) procedure that converts CSI into a fixed-length delay-domain PDP, in which the maximum resolvable delay, the delay resolution and the PDP length are identical under all bandwidths. A Transformer is then designed to exploit the fixed-size PDP input to predict the AAV location. This approach is illustrated in Figure 2.

#### 3.1.1. Zero-Padding Procedure for Bandwidth-Adaptive PDP

The ZP-PDP construction consists of three concrete stages: (i) **parameter determination:** determine a target time-domain sampling interval (delay resolution) and the corresponding IFFT length; (ii) **zero-padding:** insert zeros according to a computed oversampling factor; and (iii) **IFFT and truncation:** perform IFFT and truncate the resulting time-domain response to a fixed number of samples used as the neural network input. The detailed steps are as follows.

**(i) Parameter determination:** Let $\Delta \tau^{*}$ denote the target delay sampling interval (time-domain resolution) that we want all PDPs to share, and let $\tau_{\max}$ denote the maximum observable delay we intend to represent. The number of time-domain samples to keep is

(8) $$N_{\tau }\;=\;\left\lceil \frac{\tau_{\max}}{\Delta \tau^{*}}\right\rceil .$$

Both $\Delta \tau^{*}$ and $\tau_{\max}$ are design parameters determined by the localization range and the desired delay resolution, which remain fixed for all bandwidths.

For an IFFT of length *L* applied to frequency samples with subcarrier spacing Δf, the time-domain sampling interval, i.e., the delay resolution [29], is

(9) $$\Delta \tau \;=\;\frac{1}{L\;\Delta f}.$$

Therefore, to achieve the target resolution $\Delta \tau^{*}$ for a given subcarrier spacing Δf, the required IFFT length is

(10) $$L^{*}\;=\;\frac{1}{\Delta f\;\Delta \tau^{*}}.$$

Let the measured CSI vector at AP *n* have length *K* with subcarrier spacing Δf. The natural IFFT length without zero-padding is *K*, yielding a time resolution $\Delta \tau_{0}=1/\left(K\Delta f\right)$. To reach the target *L* we define the oversampling factor

(11) $$s\;=\;\left\lceil \frac{L^{*}}{K}\right\rceil ,$$

and set the IFFT length used in practice to

(12) L=sK.

Note that by (10) and (11) we ensure $\Delta \tau =1/\left(L^{\prime }\Delta f\right)\leq \Delta \tau^{*}$.

**(ii) Zero-padding:** According to the calculated oversampling factor *s*, we insert the frequency domain CSI with zeros to get zero-padded CSI $y_{n}^{\left(\mathrm{zp}\right)}$, which is achieved by padding s−1 zeros after each subcarrier sample. This zero-padding is written as

(13) $$y_{n}^{\left(\mathrm{zp}\right)}\left[q\right]=\left\{\begin{array}{cc} y_{n,k}, & q=s(k-1),\\ 0, & \mathrm{otherwise}. \end{array}\right.$$

**(iii) IFFT and truncation:** Compute the IFFT of length $L^{\prime }$,

(14) $$r_{n}\;=\;{\mathrm{IFFT}}_{L}\;\left(y_{n}^{\left(\mathrm{zp}\right)}\right),$$

producing a time-domain vector of length $L^{\prime }$. The PDP vector is $p_{n}={\left|r_{n}\right|}^{2}$. Then if Δτ is not exactly $\Delta \tau^{*}$, apply 1-D interpolation on $p_{n}$ with the $\Delta \tau^{*}$ spacing to get ${\bar{p}}_{n}$. Finally, we truncate the first $N_{\tau }$ samples corresponding to delays $\left[0,\;\tau_{\max}\right)$:

(15) $${\tilde{p}}_{n}\;=\;{\bar{p}}_{n}\left[\;0:N_{\tau }-1\;\right].$$

The truncated vector ${\tilde{p}}_{n}$ is the fixed-length PDP input used by the following Transformer-based neural networks.

#### 3.1.2. Transformer-Based Localization Network

We design a Transformer-based network [30] that (i) embeds each PDP to a *D*-dimensional token, (ii) injects an AP-specific index embedding, (iii) applies a multi-layer Transformer encoder operating on the AP-token sequence, and (iv) pools the output tokens and maps the pooled feature to the 3D position estimate. The self-attention mechanism allows the network to capture global dependencies across APs and delay bins, which naturally arise from the multipath structure and multi-AP geometry in localization problems. The detailed description of these modules are as follows.

**(i) PDP embedding**: Each per-AP PDP ${\tilde{p}}_{n}$ is first projected to a *D*-dimensional embedding via a learnable linear map, which is written as follows:

(16) $$u_{n}\;=\;W_{\mathrm{PDP}}\;{\tilde{p}}_{n}\;+\;b_{\mathrm{PDP}},$$

where $W_{\mathrm{PDP}}\in R^{D\times N_{\tau }}$ and $b_{\mathrm{PDP}}\in R^{D}$.

**(ii) AP index embedding:** To allow the network to distinguish tokens coming from different APs, we use a learned AP index embedding:

(17) $$e_{n}=n\cdot w_{\mathrm{idx},n}\;\;+\;b_{\mathrm{idx}},\;n=1,\ldots ,N_{\mathrm{AP}}.$$

where $w_{\mathrm{idx},n}\in R^{D}$ and $b_{\mathrm{idx}}\in R^{D}$. The input token for AP *n* is written as

(18) $$z_{n}^{\left(0\right)}\;=\;u_{n}+e_{n}.$$

**(iii) Transformer encoder with self-attention**: We use a Transformer encoder to process the PDP and AP index-embedded tokens. In general, the Transformer encoder is applied along the AP-token dimension: the input sequence length is $N_{\mathrm{AP}}$ and each token dimension is *D*. This design treats each AP’s PDP as a single informative token. For the *l*-th Transformer layer, denote the input token sequence as $Z^{\left(l-1\right)}={\left[z_{1}^{\left(l-1\right)},\ldots ,z_{N_{\mathrm{AP}}}^{\left(l-1\right)}\right]}^{T}\in R^{N_{\mathrm{AP}}\times D}.$ A multi-head self-attention (MHSA) with *H* heads block computes

(19) $$\begin{array}{cc} Q_{h}^{\left(l\right)} & =Z^{\left(l-1\right)}W_{Q,h}^{\left(l\right)},\;K_{h}^{\left(l\right)}=Z^{\left(l-1\right)}W_{K,h}^{\left(l\right)},\;V_{h}^{\left(l\right)}=Z^{\left(l-1\right)}W_{V,h}^{\left(l\right)}, \end{array}$$

(20) $$\begin{array}{cc} {\mathrm{head}}_{h}^{\left(l\right)} & =\mathrm{softmax}\;\left(\frac{Q_{h}^{\left(l\right)}K_{h}^{(l)T}}{\sqrt{d_{k}}}\right)V_{h}^{\left(l\right)},\;h=1,\ldots ,H, \end{array}$$

(21) $$\begin{array}{cc} Z_{\mathrm{att}}^{\left(l\right)} & =\mathrm{Concat}\;\left({\mathrm{head}}_{1}^{\left(l\right)},\ldots ,{\mathrm{head}}_{H}^{\left(l\right)}\right)W_{O}^{\left(l\right)}. \end{array}$$

where *H* denotes the number of attention heads, $W_{Q,h}^{\left(l\right)},\;W_{K,h}^{\left(l\right)},\;W_{V,h}^{\left(l\right)}\in R^{D\times d_{k}}$ denote the linear projection matrices for the query, key, and value of the *h*-th head and *l*-th layer, respectively, $d_{k}$ denotes the dimensionality of each head, $W_{O}^{\left(l\right)}\in R^{Hd_{k}\times D}$ denotes the output projection matrix, and $Z_{\mathrm{att}}^{\left(l\right)}$ denotes the final output of the multi-head attention module at layer *l*. The MHSA output is then added residually and layer-normalized:

(22) $${\tilde{Z}}_{\mathrm{att}}^{\left(l\right)}=\mathrm{LayerNorm}\;\left(Z^{\left(l-1\right)}+\mathrm{MHSA}\left(Z_{\mathrm{att}}^{\left(l\right)}\right)\right).$$

A feed-forward network (FFN) and layer normalization are then applied:

(23) $$Z^{\left(l\right)}=\mathrm{LayerNorm}\;\left({\tilde{Z}}_{\mathrm{att}}^{\left(l\right)}+\mathrm{FFN}\left({\tilde{Z}}_{\mathrm{att}}^{\left(l\right)}\right)\right).$$

After *M* layers we obtain $Z^{\left(M\right)}={\left[z_{1}^{\left(M\right)},\ldots ,z_{N_{\mathrm{AP}}}^{\left(M\right)}\right]}^{T}$.

**(iv) Token aggregation and localization regression:** We aggregate the output tokens into a global feature g via mean pooling:

(24) $$g\;=\;\frac{1}{N_{\mathrm{AP}}}\sum_{n=1}^{N_{\mathrm{AP}}}z_{n}^{\left(M\right)}.$$

Finally, an multi-layer perceptron (MLP) regressor maps g to the estimated 3D coordinate:

(25) $$\hat{u}\;=\;W_{\mathrm{out}}^{\left(2\right)}\;\sigma \;\left(W_{\mathrm{out}}^{\left(1\right)}g+b_{\mathrm{out}}^{\left(1\right)}\right)+b_{\mathrm{out}}^{\left(2\right)},$$

where σ(·) denotes a nonlinear activation (e.g., RELU), $W_{\mathrm{out}}^{\left(1\right)}\in R^{D^{\prime }\times D}$, $W_{\mathrm{out}}^{\left(2\right)}\in R^{3\times D^{\prime }}$, $b_{\mathrm{out}}^{\left(1\right)}\in R^{D^{\prime }}$, $b_{\mathrm{out}}^{\left(2\right)}\in R^{3}$, and $D^{\prime }$ denotes the hidden neuron numbers of the MLP.

### 3.2. Sub-Band-Sliced PDP Approach

The Sub-band-Sliced PDP (SS-PDP) approach utilizes the PDP of several equal-bandwidth sub-bands and stacks them as multiple measurement tokens. Then the Transformer-based neural network is used to predict the location.

#### 3.2.1. Sub-Band-Sliced PDP Preprocessing

**Figure 3:**
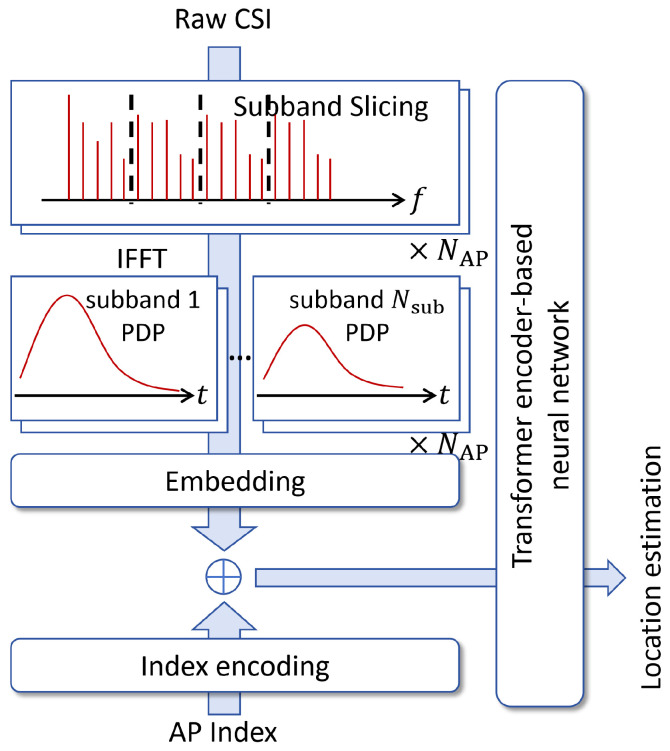
Illustration of the SS-PDP approach.

The preprocessing includes **(i) parameter determination**, **(ii) sub-band division**, **(iii) oversampling via zero-padding** and **(iv) sub-band PDP computation**. The process diagram from the raw CSI and AP index to the estimated position is shown in Figure 3. This design ensures that each sub-band’s PDP shares the same time-domain sampling interval and maximum delay coverage, while differences in overall bandwidth manifest only as a different number of sub-bands. The detailed procedure is as follows.

**(i) Parameter determination:** Choose a target sub-band width $B_{\mathrm{sub}}$, and then the number of whole sub-bands that fit into *B* is chosen by flooring:

(26) $$N_{\mathrm{sub}}\;=\;\left\lfloor \frac{B}{B_{\mathrm{sub}}}\right\rfloor .$$

We intentionally use the floor operator: any remaining partial-band at the band edges is ignored (alternatively one may distribute the remainder or zero-pad the last sub-band, but in this work we use (26) for simplicity and stability). The per-sub-band subcarrier count is written as

(27) $$K_{\mathrm{sub}}\;=\;\left\lfloor \frac{K}{N_{\mathrm{sub}}}\right\rfloor ,$$

so that the total number of used subcarriers is $K_{\mathrm{used}}=N_{\mathrm{sub}}\cdot K_{\mathrm{sub}}\leq K$. These used subcarriers are typically taken contiguously from the center-outwards or according to the system indexing convention; the remaining $K-K_{\mathrm{used}}$ edge subcarriers are ignored.

**(ii) Sub-band division:** For AP *n*, we partition the first $K_{\mathrm{used}}$ samples into $N_{\mathrm{sub}}$ contiguous blocks:

(28) $$y_{n}^{\left(s\right)}={\left[y_{n,\left(s-1\right)K_{\mathrm{sub}}+1},\;y_{n,\left(s-1\right)K_{\mathrm{sub}}+2},\;\ldots ,\;y_{n,sK_{\mathrm{sub}}}\right]}^{T},\;s=1,\ldots ,N_{\mathrm{sub}}.$$

Each vector $y_{n}^{\left(s\right)}$ corresponds to sub-band *s* for AP *n*.

**(iii) Oversampling and truncation for sub-band PDP construction**

Although each sub-band inherently provides an aligned delay resolution of $1/B_{\mathrm{sub}}$, we additionally apply a zero-padding procedure, which is similar to steps (i)–(ii) used in the ZP-PDP method, so that all sub-bands obtain a unified and customized delay resolution and maximum delay range. This helps in reducing the redundant information at large delay in PDP. Denote the target delay resolution as $\Delta \tau_{\mathrm{sub}}^{*}$ and the target maximum range as $\tau_{\max,\mathrm{sub}}$. Then use (8) to obtain the truncated points $N_{\tau ,\mathrm{sub}}$, (11) to get oversampling factor $s_{\mathrm{sub}}$ and (12) to obtain the IFFT length $L_{\mathrm{sub}}$. Then zero-pad $y_{n}^{\left(s\right)}$ as in (13) with oversampling factor $s_{\mathrm{sub}}$.

**(iv) Sub-band PDP computation:** Using IFFT, the time-domain response is expressed as follows:

(29) $$r_{n}^{\left(s\right)}\;=\;{\mathrm{IFFT}}_{L_{\mathrm{sub}}}\;\left(y_{n}^{\left(s\right)}\right).$$

Then the sub-band PDP is

(30) $$p_{n}^{\left(s\right)}\;=\;|r_{n}^{\left(s\right)}{|}^{2}.$$

Perform 1D-interpolation on $p_{n}^{\left(s\right)}$ to align the target delay resolution if necessary. Then truncate it with length $N_{\tau ,\mathrm{sub}}$ as in (15), where the final sub-band PDPs are denoted as ${\tilde{p}}_{n}^{\left(s\right)}$.

#### 3.2.2. Transformer-Based Localization Network

To process SS-PDP representations, the Transformer is adopted not only for its strong modeling capacity, but more importantly for its ability to process variable-length token sequences. This property is crucial for the proposed SS-PDP representation, where the number of sub-bands (and thus input tokens) varies with the available bandwidth. After obtaining the sub-band PDPs, we describe how these PDPs are encoded before being fed into the Transformer. Since the overall Transformer architecture has already been introduced earlier, we focus only on the token construction process and the index embeddings.

Each ${\tilde{p}}_{n}^{\left(s\right)}$ is treated as an individual measurement token. Before entering the Transformer, we apply a linear projection

(31) $$u_{n}^{\left(s\right)}=W_{\mathrm{sub}}{\tilde{p}}_{n}^{\left(s\right)}+b_{\mathrm{sub}},$$

where $W_{\mathrm{sub}}\in R^{D\times K_{\mathrm{sub}}}$ and $b_{\mathrm{sub}}\in R^{D}$. The projection ensures that each sub-band PDP is mapped into a unified *D*-dimensional embedding space. Let all sub-bands belonging to the same AP share the same AP-index embedding. The learnable embedding vector for AP *n* is obtained by (17). Then the final token embedding for sub-band *s* of AP *n* becomes

(32) $$z_{n}^{\left(s\right)}=u_{n}^{\left(s\right)}+e_{n}.$$

For *N* APs and *S* sub-bands, the final token list is stacked as follows:

(33) $$Z^{\left(0\right)}={\left[z_{1}^{\left(1\right)},\ldots ,z_{1}^{\left(S\right)},\;z_{2}^{\left(1\right)},\ldots ,z_{2}^{\left(S\right)},\;\ldots ,\;z_{N_{\mathrm{AP}}}^{\left(1\right)},\ldots ,z_{N_{\mathrm{AP}}}^{\left(S\right)}\right]}^{T}.$$

Thus the Transformer receives a total of $N_{\mathrm{AP}}N_{\mathrm{sub}}$ tokens, each of dimension *d*.

Apart from the token construction described above, the remaining architecture is identical to the previously introduced Transformer architecture. The only architectural difference between this SS-PDP method and the full-PDP method is in the input token construction and AP/sub-band stacking, and the Transformer itself is unchanged.

This tokenization strategy allows the model to adapt automatically to varying bandwidths: wider-band systems produce more sub-bands and hence more tokens, while narrow-band systems produce fewer tokens. The learned AP embeddings provide spatial context, while the Transformer aggregates multi-sub-band multi-AP delay-domain information in a bandwidth-transparent manner.

## 4. Numerical Results

### 4.1. Configurations

#### 4.1.1. Hyperparameters

The system configurations are described as follows. The number of APs is $N_{\mathrm{AP}}=11$. We consider a set of bandwidths, i.e., B∈{20, 40, 60, 80, 100}MHz and the subcarrier interval is Δf=15kHz. The AAV is located in a box which has the size of 2m×2m×8m. This region is intentionally chosen to model the AAV takeoff and landing scenario, where the vehicle operates close to the ground and experiences the most severe multipath effects. Compared with large-area scenarios, this setting represents a worst-case localization condition dominated by strong ground reflections and a dense multipath, and therefore provides a stringent stress test for the proposed method. We consider a multipath-limited regime with moderate-to-high SNR, where noise is not the dominant source of localization error. The channel dataset is generated using a ray-tracing framework, and all neural networks are trained and evaluated using the PyTorch 2.7 framework. For each bandwidth, 8000 dataset samples are generated, of which 80% are taken as the training set and 20% are taken as the testing set. The training and test sets are randomly sampled from different spatial locations in the localization space.

For the neural network hyperparameters, $\Delta \tau^{*}=0.167\;\mathrm{ns},$ $\Delta \tau_{\mathrm{sub}}^{*}=3.33\;\mathrm{ns},$ $N_{\tau }=N_{\tau ,\mathrm{sub}}=100\;\mathrm{ns},$ $D=d_{k}=D^{\prime }=256,$ M=1, and H=8. The initial learning rate is 0.001 with an exponential decay factor 0.995 per epoch. The Adam optimizer is adopted and each neural network is trained for 30 epochs.

#### 4.1.2. Baselines

To comprehensively evaluate the proposed bandwidth-adaptive localization framework, we compare it with a set of representative baselines. The baselines and the proposed methods are listed as follows.

- **Super-resolution TOAs + Least-Squares (LS)**. A classical analytic pipeline is implemented by first extracting the TOA using a super-resolution estimator, e.g., MUSIC-based delay estimation [8], from the measured channel information, followed by a geometric LS solver for position estimation.
- **DNN with super-resolution TOAs as input**. The TOA extracted by the super-resolution estimator is fed into a DNN for direct position prediction [19]. The DNN contains three hidden layers with (512, 256, 128) neurons.
- **CSI + Transformer**. A Transformer based on CSI in [23] is adopted, where only the CSI modality is used to ensure a fair comparison of different data representations and preprocessing strategies. For fairness, we adopt the same Transformer architecture as in our proposed method so that performance differences originate purely from input modalities and preprocessing rather than model capacity.
- **ZP-PDP + DNN**. Because the proposed ZP-PDP produces a bandwidth-independent, fixed-length representation, it is compatible with a DNN. The DNN contains three hidden layers with (512, 256, 128) neurons.
- **Proposed ZP-PDP + Transformer**. The proposed ZP-PDP preprocessing is fed into the dedicated Transformer encoder-based neural network.
- **Proposed SS-PDP + Transformer**. The proposed ZP-PDP preprocessing is fed into the dedicated Transformer encoder-based neural network.

### 4.2. Convergence Performance

**Figure 4:**
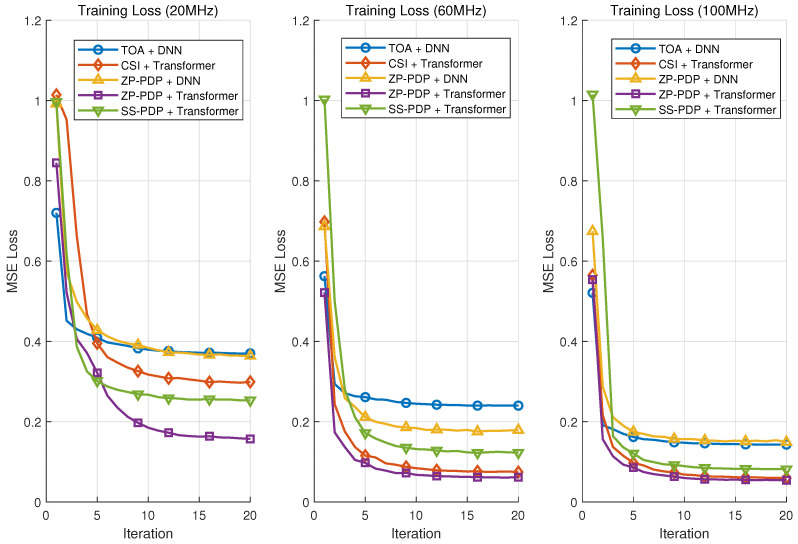
The training loss on datasets with different localization bandwidth.

Figure 4 illustrates the training loss curves obtained under different system bandwidths. The comparison covers five representative input preprocessing schemes: (i) CSI + Transformer baseline, (ii) proposed ZP-PDP + Transformer, (iii) proposed SS-PDP + Transformer, (iv) proposed ZP-PDP + DNN, and (v) TOA-based DNN baseline. Across all bandwidth configurations, the two proposed PDP-based preprocessing methods—namely the *zero-padded PDP* (ZP-PDP) with super-resolution IFFT and the *sub-band-sliced PDP* (SS-PDP)—exhibit stable and fast convergence behaviors. Their training loss trajectories closely follow, or even outperform, the CSI + Transformer counterpart. In particular, the ZP-PDP + Transformer approach consistently achieves the lowest loss among all neural models, demonstrating its superior capability in providing bandwidth-normalized delay-domain features that are highly learnable for the subsequent Transformer encoder. By contrast, the CSI + Transformer model shows clear degradation when trained using low-bandwidth (e.g., 20 MHz) data. In these cases, the reduced frequency-domain sampling leads to distorted or insufficient delay-domain information, causing instability and slower convergence. Overall, the proposed PDP preprocessing mechanisms facilitate faster and more robust optimization by providing a unified delay resolution and a structured representation across all bandwidth conditions. These characteristics enable neural networks—especially attention-based architectures—to exploit consistent temporal features, thereby achieving improved convergence and stability during training.

### 4.3. Localization Accuracy

**Figure 5:**
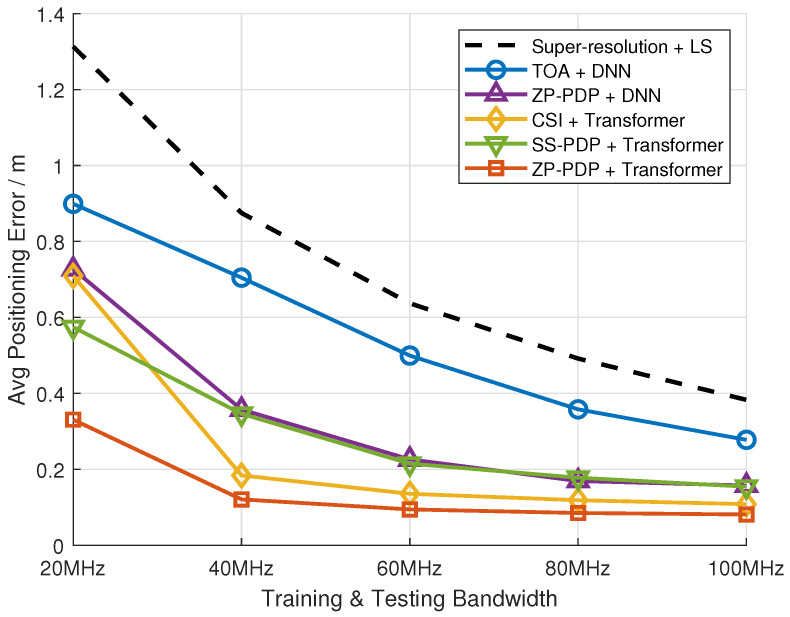
Localization error for the proposed and baseline methods.

**Figure 6:**
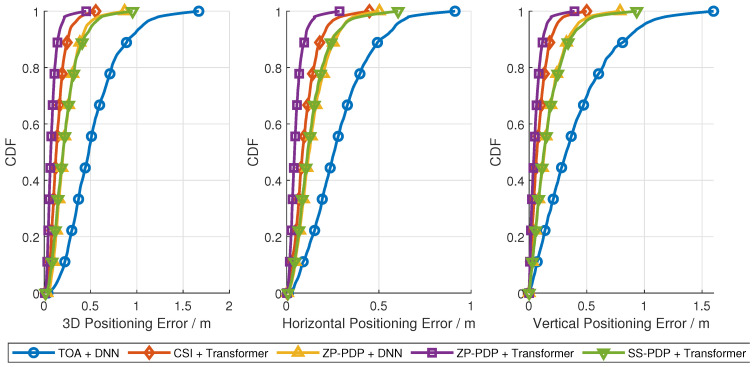
Localization error CDF at 60 MHz.

Figure 5 presents the localization errors on the test set for different system bandwidths, where the training and testing data share the same bandwidth configuration. Figure 6 presents the cumulative distribution function (CDF) of 3D, horizontal and vertical positioning errors. Table 1 presents the key percentiles of the 3D localization error CDF. In addition to neural network-based approaches, a traditional baseline method is included: super-resolution TOA estimation from wireless measurements combined with a least-squares (LS) position solver. As shown in the figure, all neural network-based methods outperform the traditional TOA + LS baseline across all bandwidths, confirming the advantage of learning-based approaches in capturing complex multipath and environmental effects. The proposed PDP preprocessing (both ZP-PDP and SS-PDP) combined with Transformer/DNN achieves comparable or superior accuracy relative to CSI + Transformer, which is consistent with the convergence analysis. This demonstrates that the PDP preprocessing retains sufficient delay-domain features for high-precision localization while normalizing the bandwidth resolution.

**Figure 7:**
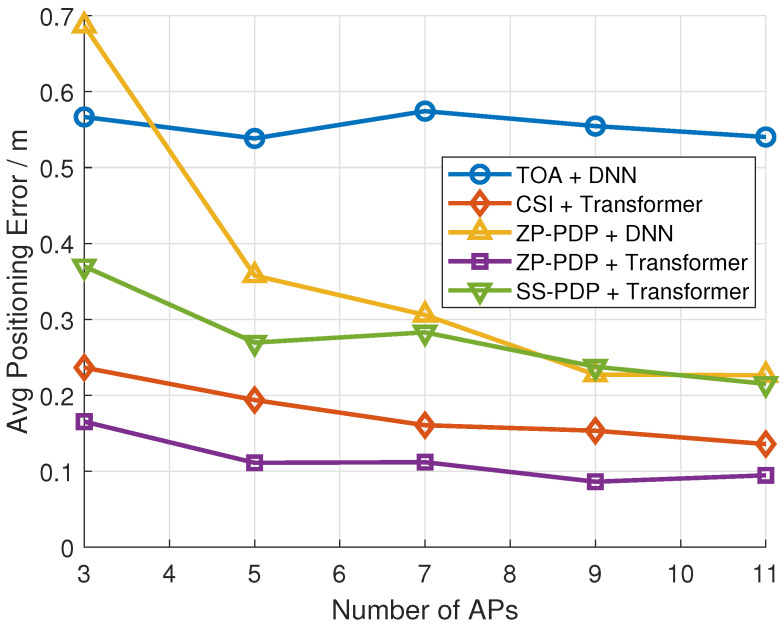
Localization error with different AP numbers at 60 MHz.

Figure 7 gives the error results applying different AP numbers. As observed, the proposed methods could take full advantage of a limited number of APs to achieve high precisions.

### 4.4. Parameter Scalability and Runtime

**Figure 8:**
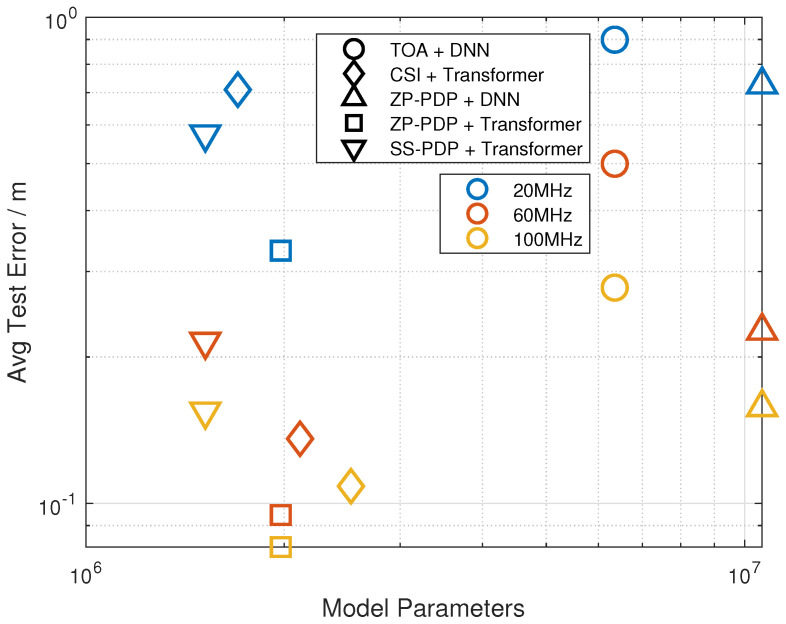
Network parameter count and localization accuracy.

Figure 8 illustrates the relationship between network parameter count and localization accuracy. A key observation is that CSI+Transformer exhibits significantly varying parameter sizes across different bandwidths, because the fully connected layers after the attention blocks must be resized to accommodate CSI vectors of different lengths. This bandwidth-dependent architectural change makes parameter sharing and model deployment across heterogeneous bandwidth configurations difficult. In contrast, the proposed PDP-based neural architectures naturally support bandwidth flexibility. For the ZP-PDP + Transformer method, the PDP resolution is normalized via zero-padding and oversampled IFFT, resulting in a fixed input dimension regardless of the system bandwidth. For the SS-PDP + Transformer method, the sub-band index is merged with the base-station dimension and treated as additional measurement tokens, allowing the Transformer to process variable numbers of sub-bands without modifying its internal structure; meanwhile, each sub-band retains clear physical meaning as an independent wide-band delay-domain observation. Furthermore, although TOA + DNN and ZP-PDP + DNN also maintain fixed input dimensions and can in principle generalize to different bandwidths, their parameter counts are notably higher while still delivering lower localization accuracy compared with Transformer-based models, indicating limited representational efficiency.

We further discuss the runtime of the proposed model. For ZP-PDP, the total computational cost is approximately 9 MFLOPs per sample, and for the maximum SS-PDP case with five sub-bands, it is about 45 MFLOPs per sample. Using the peak throughput about 11.3 TFLOPs of an NVIDIA GTX 1080 Ti and assuming a conservative 1–10% utilization, the estimated inference latency is well below 1 ms per sample.

### 4.5. Robustness Across Bandwidths

**Figure 9:**
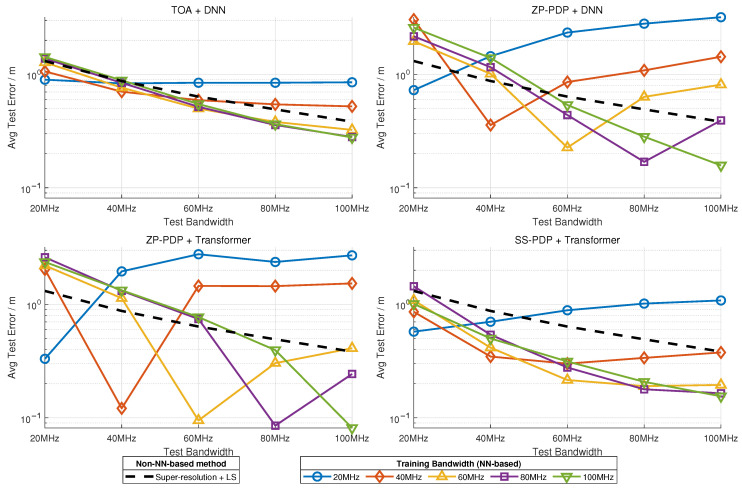
Robustness across bandwidths.

We evaluate the cross-bandwidth direct prediction performance of different methods, comparing traditional TOA-based baselines with neural approaches that allow parameter sharing across bandwidths. The results in Figure 9 show that although ZP-PDP+Transformer and ZP-PDP + DNN achieve strong or even best-in-class performance within their training bandwidth, both methods suffer from significant degradation when directly applied to unseen bandwidths. This is because, despite the consistent time scale and temporal resolution of ZP-PDP representations, changing the bandwidth alters the effective frequency support, which in turn changes the shape of the resulting PDP image and harms generalization. In contrast, the SS-PDP representation exhibits excellent cross-bandwidth robustness: its sub-band PDPs share an identical temporal scale and resolution, while differences in bandwidth merely manifest as different numbers of sub-bands, each treated as an independent statistical token, thereby preserving both interpretability and prediction accuracy. The TOA + DNN method can also operate across bandwidths, though its overall localization accuracy is noticeably lower. In summary, ZP-PDP + Transformer offers strong accuracy and consistent parameterization across bandwidths but suffers from limited bandwidth adaptability, suggesting that transfer learning or multi-bandwidth joint training could further enhance its generalization. Meanwhile, SS-PDP + Transformer naturally supports dynamic-bandwidth generalization and is particularly suitable for deployment when data collection is limited or when the operating bandwidth may vary in real time.

### 4.6. Discussions

From the convergence behavior and positioning accuracy results, the proposed PDP-based approaches achieve performance comparable to the CSI + Transformer baseline, while ZP-PDP further attains the highest accuracy. This is mainly because the ZP-PDP preprocessing enforces a unified delay scale and resolution while preserving as much measurement information as possible.

The model parameter comparison demonstrates that the model size of our method is inherently independent of the bandwidth. This contrasts with CSI-based networks, whose parameter count scales with the number of subcarriers. Therefore, the proposed framework naturally supports deployment under varying bandwidth resources.

Finally, the cross-bandwidth test results highlight the strong robustness of the proposed SS-PDP approach. Besides sharing identical delay scale and resolution across bandwidths, the uniform sub-band width ensures a consistent energy concentration pattern of the PDP. Although ZP-PDP exhibits severely degraded performance when applied to unseen bandwidths, its bandwidth-transparent architecture is well suited for low-cost adaptation through transfer learning in future extensions.

## 5. Conclusions

In this work, we have developed bandwidth-adaptive AAV localization methods based on unified delay-domain representations derived from heterogeneous CSI measurements. We have proposed two preprocessing schemes, namely ZP-PDP and SS-PDP. The ZP-PDP scheme has adaptively zero-padded and truncated CSI to form consistent PDPs with fixed delay scales and resolutions, while the SS-PDP scheme has employed sub-band slicing to ensure stable delay-domain patterns across different bandwidths. Building upon these unified representations, we have designed bandwidth-transparent Transformer encoders capable of jointly processing multi-AP measurements for accurate 3D localization. Through numerical results, we have demonstrated that ZP-PDP has achieved higher accuracy than CSI-based Transformer baseline while maintaining full bandwidth independence in model size, and that SS-PDP has exhibited superior robustness under cross-bandwidth evaluations. Overall, we have demonstrated that the integration of unified PDP preprocessing and flexible Transformer architectures has provided an effective and scalable solution for AAV localization under dynamically varying bandwidth resources. 

## Figures and Tables

**Table 1:** Key percentiles of 3D localization error CDF for different methods at 60 MHz.

Method	25%	50%	75%	90%
TOA + DNN	0.322	0.481	0.682	0.906
CSI + Transformer	0.086	0.130	0.186	0.257
ZP-PDP + DNN	0.139	0.206	0.297	0.402
ZP-PDP + Transformer	0.047	0.070	0.105	0.148
SS-PDP + Transformer	0.129	0.203	0.300	0.423

## Data Availability

The original contributions presented in this study are included in the article. Further inquiries can be directed to the corresponding author.
